# Irradiation of necrotic tumor cells used to pulse dendritic cells (DCs) potentiates DC vaccine-induced anti-tumor immunity in a mouse model of high-grade glioma

**DOI:** 10.1186/2051-1426-2-S3-P163

**Published:** 2014-11-06

**Authors:** Lien Vandenberk, Abhishek D Garg, Patrizia Agostinis, Tina Verschuere, Carolien Koks, Steven De Vleeschouwer, Stefaan Van Gool

**Affiliations:** 1 KULeuven - University of Leuven, Department of Microbiology and Immunology, Laboratory of Pediatric Immunology, B-3000 Leuven, Belgium; 2 KULeuven - University of Leuven, Department of Cellular and Molecular Medicine, Laboratory of Cell Death Research and Therapy, B-3000 Leuven, Belgium; 3 KULeuven - University of Leuven, Department of Neurosciences, Experimental Neurosurgery and Neuroanatomy, B-3000 Leuven, Belgium

## Introduction

The prognosis of high-grade glioma (HGG) is poor despite advancements in neurosurgery, radio-and chemotherapy. DC-based immunotherapy has emerged as a promising and feasible treatment approach due to its high degree of selectivity and its ability to induce an antigen-specific immune-memory response. Our research group investigates the efficacy of vaccinating both primary and relapsed HGG patients with DC vaccines pulsed with autologous whole tumor lysate. The method of preparing autologous whole tumor lysate is, however, not standardized yet with most groups applying multiple freeze-thaw (FT) cycles to induce necrosis of tumor cells. As irradiation is known to induce oxidation-associated molecular patterns (OAMPs) like oxidized or carbonylated proteins, potent enablers of danger signaling, we hypothesized that irradiation could increase the immunogenicity of FT-treated necrotic tumor cells used to pulse DC vaccines in the context of HGG.

## Methodology

All experiments were performed in the murine syngeneic GL261 orthotopic glioma model. Prophylactic DC immunotherapy was performed with murine bone marrow-derived DCs pulsed with total GL261 lysate. Total GL261 lysate was either prepared by subjecting GL261 cells to six FT cycles (GL261-FT) or by additionally applying 60 Gy X-irradiation after the six FT cycles (GL261-FT+IR).

## Results

Protein oxidation/carbonylation, an indirect measure of proteotoxicity-based immunogenicity, was significantly increased in GL261-FT+IR as compared to GL261-FT. We observed no differences in the expression levels of DC maturation markers (CD80, CD86, CD40, MHC-I and MHC-II) on matured DCs pulsed with either GL261-FT or GL261-FT+IR lysates. Flowcytometric analysis of brain-infiltrating immune cells revealed an increased infiltration of CD3+ T cells and a decreased infiltration of FoxP3+ Tregs, F4/80+ macrophages and Ly6C+ and Ly6G+ myeloid-derived suppressor cells in mice receiving prophylactic DC vaccines pulsed with GL261-FT+IR as compared to mice treated with DCs pulsed with GL261-FT. Moreover, vaccination of mice with DCs loaded with GL261-FT+IR resulted in a significant improvement of overall survival, compared to mice treated with DCs pulsed with GL261-FT.

## Conclusion

Collectively, these data suggest that irradiation of necrotic tumor cells used to pulse DC can further increase the DC vaccine-induced anti-tumor immunity in the context of glioma. We are currently evaluating the contribution of the irradiation-induced OAMPs to the increased immunogenicity of the GL261-FT+IR lysate.

